# Stable resource polymorphism along the benthic littoral–pelagic axis in an invasive crayfish

**DOI:** 10.1002/ece3.6095

**Published:** 2020-02-14

**Authors:** Iris Lang, Charlotte Evangelista, Rebecca Marie Everts, Géraldine Loot, Julien Cucherousset

**Affiliations:** ^1^ Laboratoire Évolution et Diversité Biologique (EDB UMR 5174) Université de Toulouse, CNRS, UPS Toulouse France; ^2^ Department of Biosciences Centre for Ecological and Evolutionary Synthesis (CEES) University of Oslo Oslo Norway

**Keywords:** geometric morphometrics, intraspecific variability, microsatellites, non‐native species, *Procambarus clarkii*, stable isotopes

## Abstract

Although intraspecific variability is now widely recognized as affecting evolutionary and ecological processes, our knowledge on the importance of intraspecific variability within invasive species is still limited. This is despite the fact that understanding the linkage between within‐population morphological divergences and the use of different trophic or spatial resources (i.e., resource polymorphism) can help to better predict their ecological impacts on recipient ecosystems. Here, we quantified the extent of resource polymorphism within populations of a worldwide invasive crayfish species, *Procambarus clarkii*, in 16 lake populations by comparing their trophic (estimated using stable isotope analyses) and morphological characteristics between individuals from the littoral and pelagic habitats. Our results first demonstrated that crayfish occured in both littoral and pelagic habitats of seven lakes and that the use of pelagic habitat was associated with increased abundance of littoral crayfish. We then found morphological (i.e., body and chelae shapes) and trophic divergence (i.e., reliance on littoral carbon) among individuals from littoral and pelagic habitats, highlighting the existence of resource polymorphism in invasive populations. There was no genetic differentiation between individuals from the two habitats, implying that this resource polymorphism was stable (i.e., high gene flow between individuals). Finally, we demonstrated that a divergent adaptive process was responsible for the morphological divergence in body and chela shapes between habitats while difference in littoral reliance neutrally evolved under genetic drift. These findings demonstrated that invasive *P. clarkii* can display strong within‐population phenotypic variability in recent populations, and this could lead to contrasting ecological impacts between littoral and pelagic individuals.

## Graphical abstract

Intraspecific variability is now widely recognized as affecting evolutionary and ecological processes, but few studies have to date focused on intraspecific variability within invasive species. We demonstrated that niche partitioning occurred within populations of an invasive crayfish, *Procambarus clarkii*, and that the use of the bentho‐pelagic habitat was associated with increased intraspecific competition in the littoral habitat of the studied lakes. We then found a stable resource polymorphism between littoral and pelagic crayfish, with individuals from the two habitats having different morphology and diet, but no genetic differentiation. These findings highlighted a strong within‐population variability in recent invasive populations, which could result in contrasting ecological impacts between littoral and pelagic individuals. 

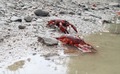

## 1 INTRODUCTION

Intraspecific variability is now widely recognized as playing a crucial role in evolutionary and ecological processes (Read, Hoban, Eppinga, Schweitzer, & Bailey, 2016; Violle et al., 2012). Genetic and/or phenotypic differences among conspecific individuals can have important implications for community structure and ecosystem functioning by mediating the intensity of bottom‐up or top‐down processes (see review in Des Roches et al., 2017; Raffard, Santoul, Cucherousset, & Blanchet, 2018). Biological invasions provide a unique opportunity to study intraspecific variability in recently established populations. Indeed, substantial trait and genetic variability among invasive individuals have been reported (Forsman, 2014; González‐Suárez, Bacher, & Jeschke, 2015), indicating that a high level of intraspecific variability can occur following the introduction stage (60–100 years after establishment, e.g., Hendry, Wenburg, Bentzen, Volk, & Quinn, 2000; Kinnison, Unwin, Boustead, & Quinn, 1998; Lankau, 2012). Because intraspecific variability can modulate the ecological effects of invasive individuals on ecosystem processes (Evangelista, Lecerf, Britton, & Cucherousset, 2017), quantifying the extent of intraspecific variability in invasive species, notably within populations and across the invasion landscape, is therefore relevant for both applied and theoretical perspectives.

**Figure 1:**
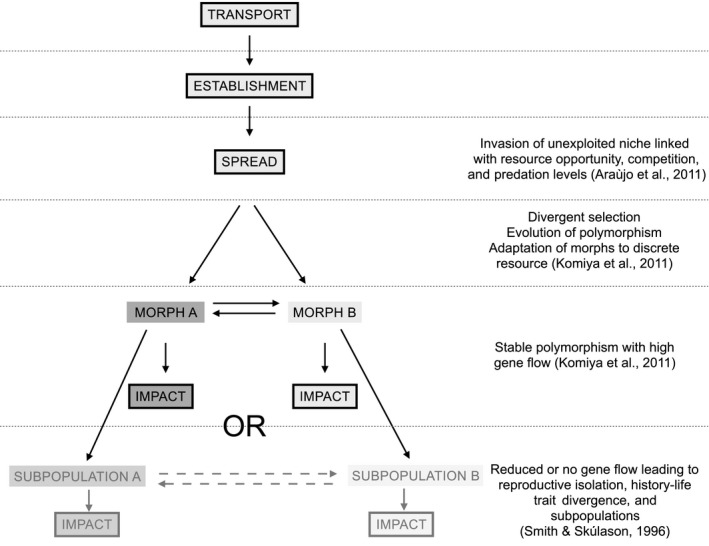
Conceptual diagram of the establishment of resource polymorphism during the different stages of a biological invasion. Resource polymorphism might result in stable resource polymorphism or in the existence of distinct subpopulations. Adapted from Smith and Skúlason (1996) and Lockwood, Hoppes, and Marchetti (2007)

Resource polymorphism refers to within‐population morphological divergences due to differences in habitat and trophic resource use (Smith & Skúlason, 1996). It involves the use of an underexploited ecological niche by some individuals of the population, associated with changes in functional traits due to new environmental conditions (Komiya, Fujita, & Watanabe, 2011; Sol et al., 2005). Stable resource polymorphism is defined as the existence of discrete morphs with no genetic isolation and is associated with high gene flow between morphs (Smith & Skúlason, 1996). When gene flow is limited, subsequent genetic isolation can occur among morphs, resulting in distinct subpopulations (Smith & Skúlason, 1996) (Figure 1). In freshwater lentic ecosystems, resource polymorphism commonly occurs along the littoral–pelagic axis (Faulks, Svanbäck, Eklöv, & Östman, 2015; Quevedo, Svanbäck, & Eklöv, 2009). Littoral and pelagic habitats have distinct environmental characteristics (e.g., resource diversity, predation pressure, habitat structure, competition), and individuals using these distinct habitats often display significant morphological differences associated with trophic niche partitioning (Bartels, Hirsch, Svanbäck, & Eklöv, 2012; Faulks et al., 2015; Marklund et al., 2019; Svanbäck, Eklöv, Fransson, & Holmgren, 2008). Additionally, genetic differences between littoral and pelagic morphs can occur due to assortative mating (Robinson & Wilson, 1996). Studying resource polymorphism could therefore provide new insights into an underexplored aspect of phenotypic variability within freshwater invasive species (but see Davidson, Jennions, & Nicotra, 2011; Huey, Gilchrist, & Hendry, 2005; Komiya et al., 2011; Yonekura, Nakai, & Yuma, 2002), which can subsequently help understanding their ecological impacts.

In the present study, we quantified the extent of resource polymorphism in populations of highly invasive red‐swamp crayfish (*Procambarus clarkii*) across bentho‐littoral and bentho‐pelagic habitats (hereafter referred as littoral and pelagic habitats). In lakes, *P. clarkii* has been reported to preferentially occupy littoral habitats (Gherardi & Acquistapace, 2007), but has also been occasionally reported in the pelagic habitat (Foster & Harper, 2006). We first aimed at quantifying the existence of variability in habitat use (littoral vs. pelagic) and at identifying its associated ecological determinants. We predicted that crayfish abundance in the pelagic habitat would increase with increased abundance of crayfish in the littoral habitat, decreased habitat availability (proportion of littoral habitat compared to proportion of pelagic habitat), increased time of invasion, and decreased predation pressure. Then, we quantified morphological and trophic traits of individuals from the littoral and pelagic habitats. We predicted the existence of differences in body and chelae morphology functionally associated with differences in habitat structure and resources consumed (trophic position and origin of resource use). Finally, we quantified genetic differentiation between individuals from the littoral and pelagic habitats to determine the stability of resource polymorphism and its underlying mechanisms (i.e., adaptive or nonadaptive processes). We predicted that gene flow would be high (i.e., associated with stable polymorphism, Smith & Skúlason, 1996) in these recently colonized ecosystems and that phenotypic variability would be mainly caused by an adaptive response to environmental conditions.

## 2 MATERIAL AND METHODS

### 2.1 Study system and model species

The study was conducted in 16 gravel pit lakes ranging from 0.7 to 27.1 ha and located along the Garonne River in southwestern France (Alp, Cucherousset, Buoro, & Lecerf, 2016; Jackson et al., 2017) (Appendix S1). Created between 1963 and 2007, these lakes are characterized by different environmental conditions arising from various levels of maturity and management practices (Zhao, Grenouillet, Pool, Tudesque, & Cucherousset, 2016). Native from Northern America, *P. clarkii* is one of the most invasive crayfish species worldwide (Oficialdegui et al., 2019). The species was introduced to France in 1976, and its presence in the studied area was first documented in 1995 (Changeux, 2003), indicating that the colonization process is relatively recent in those lakes. In the study area, *P. clarkii* are usually observed very rapidly once the lakes are created. Consequently, we assumed that lakes created before 1995 were colonized by *P. clarkii* in 1995 and that the lakes created afterward were colonized during the first year of their creation (Appendix S1). *P. clarkii* is known to induce strong negative impacts on native organisms and ecosystem processes due to predation, high competitiveness, disease transmission, and ecological engineering (Gherardi & Acquistapace, 2007; Jackson et al., 2014). Chelae are important organs involved in multiple ecological functions of crayfish (e.g., predator–prey and competitor interactions, feeding behavior, biological engineering; Gherardi, Acquistapace, & Barbaresi, 2000; Matsuzaki, Usio, Takamura, & Washitani, 2009) and are known to display intraspecific morphological variations (Claussen, Gerald, Kotcher, & Miskell, 2008; Malavé, Styga, & Clotfelter, 2018). In the studied system, previous investigations have revealed the existence of intraspecific variability among *P. clarkii* populations in terms of body morphology (Evangelista, Cucherousset, Olden, & Lecerf, 2019), trophic ecology (Jackson et al., 2017), and ecosystem impacts (Alp et al., 2016; Evangelista, Lecerf, & Cucherousset, 2019), as well as the presence of within‐population phenotypic variability (Raffard et al., 2017).

### 2.2 Sampling and environmental characteristics

*Procambarus clarkii* were sampled in the littoral and pelagic habitats of each lake. The littoral habitat was shallow (<3 m) and characterized by a high level of structural heterogeneity. The nearshore substrate was composed of a mixture of gravels and cobbles with vegetation debris (e.g., downed trees, branches, helophytes) which provided sheltering opportunities for crayfish to hide against predators. The pelagic habitat was deeper and structurally more homogeneous. The substrate was soft and exclusively composed of mud. Importantly, these lakes are not stratified.

Sampling was performed from mid‐September to mid‐October 2014 in the two habitats of each lake using pairs of baited traps (one cylindrical trap: 62 cm × 34 cm × 34 cm, mesh size: 10 mm; one rectangular trap: 95 cm × 20 cm × 20 cm, mesh size: 4 mm) set overnight (*n*_littoral_ = 4.0 traps ± 0.0 *SD*; *n*_pelagic_ = 3.9 ± 0.5 *SD*) and during the day (*n*_littoral_ = 6.0 ± 0.0 *SD*; *n*_pelagic_ = 4.9 ± 2.4 *SD*). Littoral traps were located within the first 5 m along the shoreline in a shallow part (depth mean = 1.44 m ± 0.28 *SD*). Pelagic traps were located in the central (mean distance to shoreline = 71.01 m ± 26.57 *SD*) and profundal (depth mean = 3.59 m ± 1.24 *SD*) part of each lake (Appendix S1). In each lake, we aimed at collecting 20 individuals from each habitat to capture intraspecific variability in the studied phenotypic traits (Faulks et al., 2015; Lostrom et al., 2015; Weese, Ferguson, & Robinson, 2012). When required, additional trapping in both habitats and hand netting along the shoreline (not feasible in the pelagic habitat) were performed to capture the targeted number of individuals. Crayfish were sexed, measured for carapace length (±0.01 mm), and placed on ice for anesthesia. A small sample of muscle from the abdomen was subsequently collected on each specimen, stored in RNAlater^©^, and frozen at the laboratory (−20°C) until subsequent genetic analyses. After collecting muscle tissue, each individual was placed in a labeled plastic bag and frozen in the laboratory. After defrosting, a sample of abdominal muscle was collected on each specimen, rinsed with distilled water, and oven‐dried (60°C for 48 hr) for stable isotope analyses.

On the same day as crayfish sampling, putative trophic resources of *P. clarkii* were collected in three different locations in each habitat of each lake to capture potential spatial heterogeneity in their stable isotope values. Specifically, periphyton and leaf litter were collected from the littoral, as they represent important components of *P. clarkii's* diet (Alp et al., 2016; Jackson et al., 2017), while pelagic zooplankton was collected using a 200‐µm mesh net as we assume that pelagic individuals on muddy bottoms could consume detritus including zooplankton debris (Ruokonen, Kiljunen, Karjalainen, & Hämäläinen, 2012; Smart et al., 2002). Periphyton and zooplankton samples were freeze‐dried (−50°C for 5 days) and oven‐dried (60°C for 48 hr), respectively (further details available in Jackson et al., 2017). Samples of crayfish and putative prey were collected in September–October (i.e., at the end of the growing season) to ensure that stable isotope analyses were representative of the trophic interactions occurring during this period.

The abundances of littoral and pelagic crayfish were calculated as the number of crayfish trapped over a 24‐hr period in each habitat (catch per unit effort (CPUE) expressed in ind. trap^−1^.hr^−1^, Appendix S1). On the same day, fish community was sampled to assess predation pressure. Gillnets were set in the littoral (length: 20 m, height: 2.4 m; mesh size: 12, 20, 30, 60 mm, *n* = 4 to 6 depending on the lake size) and pelagic habitats (length: 25 m, height: 3.1 m; mesh size: 20 and 50 mm, respectively, *n* = 2) following Zhao et al. (2016). Fish species were determined, and each specimen was measured for fork length (±0.01 mm). For each fish species, the body mass of each fish was computed using length–weight relationships (T. Zhao, unpublished data). Predator biomass in each lake was then calculated as the biomass of predator fish trapped in gillnets over a 1‐hr period biomass (biomass per unit effort (BPUE) expressed in g. gillnet^−1^.hr^−1^; Appendix S1). Based on gape limitation and knowledge about trophic interactions in these studied lakes, potential crayfish predators were juveniles and adults of pike *Esox lucius* (>275 mm FL), common carp *Cyprinus carpio* (all individuals), European perch *Perca fluviatilis* (>110 mm FL), pikeperch *Sander lucioperca* (>200 mm FL), largemouth bass *Micropterus salmoides* (>105 mm FL—fork length), and European catfish *Silurus glanis* (>200 mm FL). Because the studied lakes were relatively small and these predatory species are highly mobile (i.e., they feed on crayfish in both habitats; Garvey, Rettig, Stein, Lodge, & Klosiewski, 2003), a global predation pressure was calculated for each lake. In four lakes (i.e., 25% of the studied lakes), *P. clarkii* coexisted with the invasive spiny‐cheek crayfish (*Faxonius limosus*) which was present in low density (ind.trap^−1^.hr^−1^ mean = 0.07 ± 0.05 *SE*). As this species was rare, we did not consider potential interspecific competition with *P. clarkii* as a key driver of their habitat use in our analyses. The surfaces of littoral (<3 m deep) and pelagic (>3 m deep) habitats were measured for each lake using bathymetry data (Appendix S1). A depth threshold of 3.0 m was used to separate littoral from pelagic habitats following Garvey et al. (2003) and Ruokonen et al. (2012). The proportion of littoral habitat (%) was then calculated as the ratio of littoral habitat surface and total lake surface.

### 2.3 Morphological and stable isotope analyses

Each crayfish and its right chela were photographed dorsally directly after defrosting and before the tissue sample was taken for stable isotope analyses. Pictures were analyzed for morphological variations using TpsDig2 v.2.17 (Rohlf, 2015). We used a geometric morphometric technique (Zelditch, Swiderski, & Sheets, 2012) based on landmark analysis that has been widely used to quantify shape variations of morphological structures along the littoral–pelagic axis (Bartels et al., 2012; Faulks et al., 2015; Quevedo et al., 2009). Here, we digitized 19 homologous landmarks on *P. clarkii* individual bodies (i.e., cephalothorax and abdomen) following Evangelista, Cucherousset, et al. (2019) and 7 landmarks on their chela propodus (adapted from Malavé et al., 2018). For each morphological structure (i.e., body and chela), a full Procrustes fit (FPF) was then performed using Morpho J v.1.06d to obtain a global shape comparison by superimposing individual shapes and removing the bias due to different sizes, positions, and orientations among individuals (Klingenberg, 2011). The deformation components (i.e., landmark coordinates) obtained with each FPF were projected into two separate matrices to characterize whole‐body and whole‐chela shape using partial warps (i.e., nonuniform variation localized to particular regions of geometry) and uniform scores (i.e., uniform variation throughout the body or the chela) (Zelditch et al., 2012). Using whole‐body and whole‐chela datasets, the centroid size of each individual morphological structure was also calculated as the square root of the summed squared distances of each landmark from their centroid and used as a proxy of individual body and chela size.

Stable isotope samples were ground to a fine powder and analyzed for carbon (δ^13^C) and nitrogen (δ^15^N) stable isotopes at the Cornell Isotope Laboratory (COIL). The trophic position (TP_crayfish_) of each individual was computed following Vander Zanden, Cabana, and Rasmussen (1997):

$${\text{TP}}_{\text{crayfish}}={\text{TP}}_{\text{baseline}}+\left(\delta^{15}N_{\text{crayfish}}-\delta^{15}N_{\text{baseline}}\right)/3.4$$

where baseline organisms are a mix of leaf litter (allochthonous primary producer) and periphyton (autochthonous primary producer) (TP_baseline_ = 1), δ^15^N_baseline_ corresponds to the mean of δ^15^N_periphyton_ and δ^15^N_litter_, and 3.4 is the fractionation coefficient between trophic levels (Post, 2002; Vander Zanden et al., 1997). The origin of resource use was assessed by quantifying the littoral reliance (LR: relative dietary contribution of littoral resources to each individual), with periphyton and zooplankton as baselines for littoral and pelagic habitats, respectively, and following Vander Zanden & Vadeboncoeur (2002):

$${\text{LR}}_{\text{crayfish}}=\left(\delta^{13}C_{\text{crayfish}}-\delta^{13}C_{\text{zooplankton}}\right)/\left(\delta^{13}C_{\text{periphyton}}-\delta^{13}C_{\text{zooplankton}}\right).$$

Regarding littoral reliance, zooplankton is the only group of primary consumers that was consistently collected in all studied lakes, and which could contributed to the diet of crayfish (Alcorlo, Geiger, & Otero, 2004; Correia, 2003). We have considered that *P. clarkii* were not selective on zooplankton taxa, so the pooled samples have been analyzed even though zooplankton have varying trophic positions (Matthews & Mazumder, 2003).

### 2.4 Genetic analyses

Neutral genetic differentiations were assessed using 14 microsatellites. Ten microsatellites were selected from Belfiore and May (2000) (PclG04, PclG07, PclG15, PclG16, PclG17, PclG27, PclG28, PclG29, PclG32, and PclG48) and 4 additional microsatellites (PCSH0038, PCSH0005, PCSH0006, and PCSH0089) were used, based on Jiang et al. (2015). DNA was extracted from the abdomen muscle of crayfish using a salt‐extraction method (Aljanabi & Martinez, 1997). Loci were amplified by polymerase chain reaction (PCR) in a final volume of 10 μl, containing 10–20 ng of genomic DNA, 5 μl of QIAGEN, and locus‐specific optimized combination of primers (see Appendix S2). PCR was performed in a Mastercycler (Eppendorf®) under the following conditions: 15 min at 95°C followed by 35 cycles of 0.5 min at 94°C, 1.5 min at 56°C, and 1 min at 72°C, and finally followed by a 45 min final elongation step at 60°C (see Appendix S2 for the description of the multiplex used in this study). Amplified fragments were analyzed on an ABI PRISM 3730 capillary sequencer (Applied Biosystems) in the Génopole Toulouse Midi‐Pyrénées. Allele size results were scored using GENEMAPPER v.4.0 (Applied Biosystems). Then, deviations from Hardy–Weinberg equilibrium (HWE) and linkage disequilibrium (LD) between all pairs of loci were tested using FSTAT v.2.9.3.2 (Goudet, 2002) and null alleles were tested using MICROCHECKER v.2.2.3 (Van Oosterhout, Hutchinson, Wills, & Shipley, 2004). Genetic diversity was quantified using observed (Ho) and expected (He) heterozygosity, Wright fixation indices (*F*_IS_) and allelic richness (A_R_) are based on the minimum sampling size. The genetic differentiation (*F*_ST_) between littoral and pelagic individuals was calculated using FSTAT v.2.9.3.2 (Goudet, 2002). No null alleles were detected in the genotyped loci. The number of alleles per locus ranged from 2 to 11 (Table 1). We found no linking disequilibrium between pairs of loci. There was no evidence for any significant heterozygous deficit for the considered loci after Bonferroni correction, suggesting that all populations were at Hardy–Weinberg equilibrium.

**Table 1:** Genetic diversity of *Procambarus clarkii* in the littoral and pelagic habitats of 7 studied lakes based on 14 microsatellites

Lake	Habitat	NA	A_R_	He	Ho	*F*_IS_
A	Littoral	5.0714	4.8065	0.6585	0.6821	−0.010
	Pelagic	5.1429	4.9456	0.6709	0.7120	−0.033
B	Littoral	4.7143	4.3966	0.5921	0.5918	0.027
	Pelagic	4.6429	4.4206	0.6102	0.5523	0.122
G	Littoral	6.4286	5.9626	0.7014	0.7297	−0.014
	Pelagic	6.4286	6.0222	0.6898	0.6582	0.073
I	Littoral	4.8571	4.6441	0.6407	0.6172	0.064
	Pelagic	4.4286	4.1959	0.6181	0.6417	−0.013
J	Littoral	5.7857	5.4438	0.6897	0.6910	0.026
	Pelagic	6.0000	5.8495	0.7127	0.6964	0.056
K	Littoral	5.1429	4.7662	0.6480	0.6679	−0.005
	Pelagic	5.2143	4.7738	0.6461	0.6455	0.027
M	Littoral	3.4286	3.3727	0.5616	0.5438	0.058
	Pelagic	3.7143	3.6164	0.5882	0.6212	−0.030

Abbreviations: A_R_, allelic richness; *F*_IS_, fixation indices; He, expected heterozygosity; Ho, observed heterozygosity; NA, mean number of alleles per locus.

### 2.5 Statistical analyses

To test the association between environmental conditions and the abundance of *P. clarkii* in the pelagic habitat of the 16 lakes, we used a linear model (LM) with abundance of *P. clarkii* in littoral habitat, predation pressure (predators' biomass), habitat availability (proportion of littoral habitat), time of invasion (expected date of lake invasion), and their two‐way interactions as fixed effects. The full model was built, and interactions were removed when nonsignificant using a backward procedure. Variance inflation factor (VIF) did not detect multicollinearity between predictors (VIF < 5) (R package “car” v. 3.0‐0; Fox & Weisberg, 2018).

Comparison of ecological and genetic characteristics between habitats was performed in 7 lakes where a sufficient number of individuals were collected in each habitat (mean number of individuals per habitat = 19.79 ± 0.8 *SD*; Appendix S1). We used a linear mixed model (LMM) with “carapace length” as response variable, “habitat” and “sex” as fixed effects, and “lake” as random effect to test for a significant difference of crayfish size between littoral and pelagic individuals. To remove potential allometric component of shape variation, partial warps scores were regressed against centroid sizes, using a pooled within‐habitat regression in Morpho J (Klingenberg, 2016). Regression residual scores (for body and for chela) were analyzed in two distinct discriminant function analyses (DFA) implemented in Morpho J to determine whether the morphology of individuals from the littoral and pelagic habitats differed significantly (Klingenberg, 2011). Each individual was thus characterized by a DFA morphological score for both body and chela along the littoral–pelagic axis. Sexual dimorphism is a potent agent of intraspecific morphological variability (Malavé et al., 2018), and this effect was assessed using a LMM with “DFA score” as response variable, “sex” and “habitat” and their interaction as fixed effects, and “lake” as random effect. When significant, the interaction was further investigated with post hoc pairwise comparison of the estimated marginal means using the “emmeans” function in the “emmeans” R package v.1.4.3.01 (Lenth, 2019). Because trophic resources’ use has been reported not to differ between sex in crayfish (Gutiérrez‐Yurrita et al., 1998; Houghton, Wood, & Lambin, 2017; Pérez‐Bote, 2004), sex was not included in the subsequent trophic analyses. To test for the presence of resource polymorphism, we first used a LMM with “trophic position” as response variable and a generalized linear mixed model (GLMM) with “littoral reliance” as response variable, with each model including “habitat” and “lake” as fixed and random effects, respectively. The LMMs were run using “lme4” R package v. 1.1‐21 (Bates, Maechler, Bolker, & Walker, 2015). Littoral reliance was a continuous variable bounded between 0 and 1, and the GLMM was thus run using a beta distribution in the “glmmTMB” R package v. 0.2.3 (Brooks et al.,^2017^). We then tested the association between individual morphology (body and chela DFA scores) and origin of resource use (i.e., littoral reliance) using a beta regression implemented in the “betareg” R package v3.1‐1 (Cribari‐Neto & Zeilis, 2010). For all models, residual normality and homoscedasticity were checked using Q‐Q plot and Tukey–Anscombe plot, respectively. Abundance of *P. clarkii* in pelagic habitat was square‐root‐transformed to conform with these assumptions.

Genetic and phenotypic differentiations were compared to determine the underlying process (neutral or adaptive process) explaining variability between littoral and pelagic individuals (Leinonen, Cano, Mäkinen, & Merilä, 2006). We used a quantitative genetic approach based on *F*_ST_ calculated using microsatellites as neutral genetic markers, and the phenotypic equivalent *P*_ST_ (i.e., used as a proxy of *Q*_ST_ for natural environments) was calculated for both morphology and diet as:

$$P_{{\text{ST}}_{X}}=\sigma_{\text{betweenpops}}^{2}/\left(\sigma_{\text{betweenpops}}^{2}+2h^{2}\sigma_{\text{withinpop}}^{2}\right)$$

where *σ* ^2^ is the variance of the phenotypic trait *X* (i.e., DFA scores, trophic position, or littoral reliance) and *h* ^2^ is the heritability of *X* defined as the proportion of phenotypic variance with a genetic origin (Leinonen et al., 2006). Because we had no information on trait heritability, *h* ^2^ was set to 0.5 to avoid overestimating *P*_ST_ (e.g., heritability estimates for the studied traits are close to 0.3–0.5; Lutz & Wolters, 1989). When *P*_ST_ and *F*_ST_ are equal, considered traits evolve neutrally under genetic drift. A greater *P*_ST_ than *F*_ST_ implies an adaptive phenotype divergence, while a higher *F*_ST_ suggests a homogenizing adaptation. We estimated the between‐habitats variance using LMMs (“lme4” R package v. 1.1‐21) with the phenotypic traits as response variables, the intercept as a fixed effect, and the “habitat” as a random effect. “Littoral reliance” was square‐root‐transformed to improve the fit of the model. We computed 95% confidence interval (CI 95%) for *P*_ST_, using bootstrapping procedure (Raffard et al., 2019), while CI 95% for *F*_ST_ was implemented in FSTAT. All statistical analyses were performed using R v.3.4.4 (R Core Team, 2018).

## 3 RESULTS

*Procambarus clarkii* occurred in all littoral habitats, and was detected in the pelagic habitat of 75% of the sampled lakes. *P. clarkii* abundances within littoral and pelagic habitats were highly variable among lakes, ranging from 0.00 to 4.79 ind. trap^−1^.hr^−1^ in the littoral habitat and from 0.00 to 10.63 ind. trap^−1^.hr^−1^ in the pelagic ones (Appendix S1). In the latter, crayfish abundance was significantly and positively related to the abundance of *P. clarkii* in the littoral habitat (LM, *F*_1,10_ = 38.90, *p* < .001). There was no significant effect of the proportion of littoral habitat (LM, *F*_1,10_ = 0.49, *p* = .500), predators’ biomass (LM, *F*_1,10_ = 0.04, *p* = .856), and time of invasion (LM, *F*_1,10_ = 2.87, *p* = .121) on the abundance of *P. clarkii* in the pelagic habitat.

**Figure 2:**
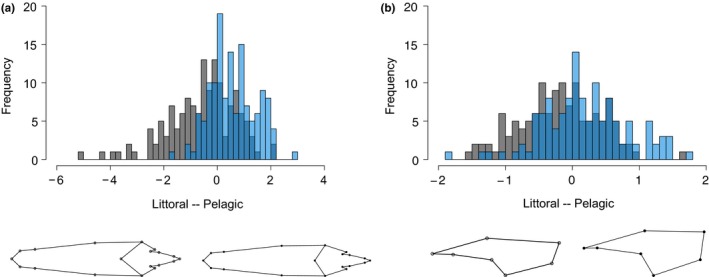
Frequency distribution of the morphological scores of *Procambarus clarkii* obtained using a discriminant functional analysis (DFA) between littoral (dark gray) and pelagic (blue) habitats for (a) bodies (*n* = 140 and *n* = 137, respectively) and (b) chelae (*n* = 129 and *n* = 124, respectively). Body and chela shapes of extreme landmark‐based values are displayed (amplified ten times)

**Figure 3:**
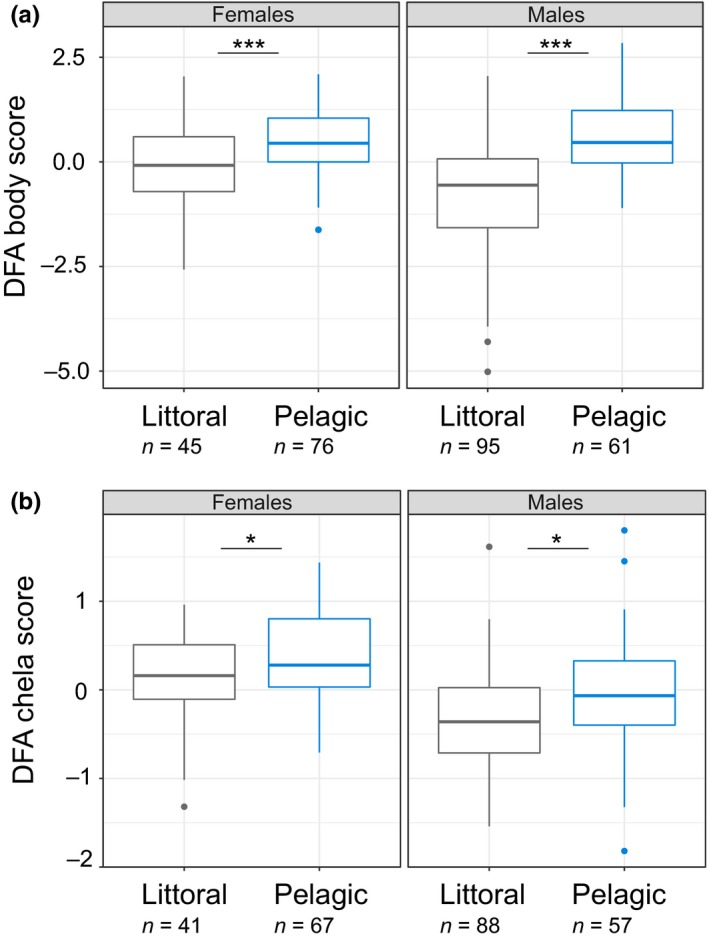
Boxplot of DFA scores for (a) bodies (*n*_females_ = 121 and *n*_males_ = 156) and (b) chelae (*n*_females_ = 108 and *n*_males_ = 145) for female and male individuals captured in littoral (dark gray) and pelagic (blue) habitats

**Figure 4:**
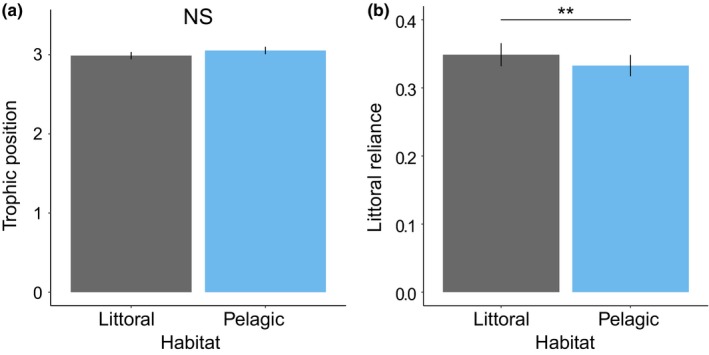
Trophic position (a) and littoral reliance (b) of *Procambarus clarkii* in littoral (dark gray; *n* = 139) and pelagic (blue; *n* = 137) habitats. Predicted values obtained from the models are reported with their standard errors

In the 7 lakes where *P. clarkii* was abundant in both littoral and pelagic habitats, sex ratio did not differ between littoral and pelagic habitats (*t* test, *t* = 2.23, *df* = 6, *p* = .068; performed using all the crayfish captured). Carapace lengths differed significantly between females and males (LMM, *F*_1,268_ = 8.89, *p* = .003), with females displaying longer carapace lengths than males (mean ± *SE* = 49.14 ± 0.50 mm and 47.62 ± 0.41 mm, respectively). There was no significant difference in carapace length between individuals from littoral and pelagic habitats (LMM, *F*_1,268_ = 0.65, *p* = .423; Appendix S3). However, individuals from each habitat differed significantly in body morphology (DFA; *T*‐square = 80.31; *p* < .001). Specifically, individuals from littoral habitat had lower DFA scores than those from pelagic habitat (mean = −0.06 ± 0.11 *SE* and 0.62 ± 0.08 *SE*, respectively), indicating that littoral crayfish were characterized by stocky body and rostrum, while pelagic crayfish had a more streamlined body and rostrum (Figure 2a). It is, however, interesting to note that the extent of morphological differences between littoral and pelagic individuals varied between lakes (Appendix S4). Even if females had higher DFA body scores than males (mean = 0.30 ± 0.09 *SE* and −0.24 ± 0.11 *SE*, respectively), the effect of sex alone did not affect body shape. However, DFA scores were significantly affected by the effect of habitat (LMM, *F*_1,268_ = 79.16, *p* < .001; as also seen with the DFA) and this effect was sex‐dependent (LMM, interaction term: *F*_1,270_ = 18.54, *p* = .004). Specifically, for both sexes, pelagic individuals had higher DFA body scores compared to littoral ones (post hoc tests, *t* ratio = −3.62, *df* = 268, *p* < .001 and *t* ratio = −8.59, *df* = 269, *p* < .001 for females and males, respectively; Figure 3a). Chela morphology also differed significantly between littoral and pelagic individuals (DFA; *T*‐square = 23.7; *p* = .014). Individuals from littoral habitat displayed lower DFA scores than those from pelagic (mean = −0.19 ± 0.05 *SE* and 0.19 ± 0.06 *SE*, respectively), indicating that chelae from littoral individuals were thicker while those from pelagic individuals were more elongated (Figure 2b). The effect of habitat on chela shape was not sex‐dependent (LMM, interaction term: *F*_1,246_ = 0.0035, *p* = .953) but DFA chela scores also differed significantly between females and males (LMM, *F*_1,248_ = 43.56, *p* < .001), with females displaying higher DFA chela scores than males (mean = 0.39 ± 0.05 *SE* and −0.23 ± 0.05 *SE*, respectively; Figure 3b). Trophic position of *P. clarkii* did not significantly differ between littoral (mean = 3.00 ± 0.05 *SE*) and pelagic (mean = 3.05 ± 0.05 *SE*) individuals (LMM, *F*_1,268_ = 2.96, *p* = .086; Figure 4a, Appendix S5). However, littoral reliance of littoral individuals (mean = 0.35 ± 0.02 *SE*) was significantly higher than for pelagic individuals (mean = 0.33 ± 0.02 *SE*), supporting the existence of a differential niche use (GLMM, χ^2^ = 7.50, *df* = 1, *p* = .006; Figure 4b, Appendix S5).

There was no significant genetic differentiation between individuals from the littoral and pelagic habitats (littoral–pelagic global *F*_ST_ = 0.000, CI 95%: 0.000–0.002 and ANOVA – He: *p* = .407; A_R_: *p* = .561; Table 1). *P*_ST_ for body morphology and chela morphology was 0.364 (CI 95%: 0.269–0.457) and 0.152 (CI 95%: 0.056–0.275), respectively, and was significantly higher than *F*_ST_, indicating that morphological variations were due to adaptive divergent processes. *P*_ST_ for trophic position and littoral reliance was 0.000 (CI 95%: 0.000–0.0448) and 0.000 (CI 95%: 0.000–0.0542), respectively. There was no significant difference between *P*_ST_ for trophic position and littoral reliance and the global *F*_ST_ value, indicating that both trophic traits evolved under nonadaptive processes (i.e., genetic drift).

## 4 DISCUSSION

Our results supported the existence of resource polymorphism within invasive species. Indeed, we observed that morphological divergences between littoral and pelagic habitats were also associated with changes in the origin of the resource used by crayfish. This resource polymorphism might occur due to intraspecific competition since the abundance of pelagic crayfish was strongly and positively associated with the abundance of littoral crayfish. There was no genetic differentiation between individuals from the two habitats, indicating that the resource polymorphism was stable (Figure 1). Finally, we demonstrated that morphological divergences in body and chela shapes between habitats were driven by a divergent adaptive process, while differences in littoral reliance neutrally evolved under genetic drift.

Although crayfish abundance was highly variable between lakes, the species occurred in the pelagic habitat of 75% of the studied lakes and was abundant in both littoral and pelagic habitats in 44% of the studied lakes. In addition, we found that increased pelagic abundance was associated with increased littoral abundance. This suggests that intraspecific competitive exclusion was a potential mechanism explaining the presence of crayfish in the pelagic habitat. Increased population density in the preferred littoral habitat of crayfish (Nyström et al., 2006) can favor aggressiveness between conspecifics (Gherardi & Cioni, 2004), limiting the access to shelters (e.g., under cobble, tree trunks, macrophytes, rocks) and forcing some weak competitors to migrate to the pelagic habitat. Competitive exclusion may also explain morphological divergences between littoral and pelagic crayfish for both sexes, with stockier‐bodied and streamlined individuals occupying the littoral and pelagic habitats, respectively. Although this remains to be tested experimentally, individuals with a stocky cephalothorax and rostrum and with longer chelae might have a competitive advantage compared to more streamlined individuals to occupy littoral shelters (Stein, 1977).

Predation might also be a driver of morphological differences observed between the two habitats (Kershner & Lodge, 1995; Stein, 1977). In the littoral habitat, stockier‐bodied individuals might have an advantage to face predation pressure from both aquatic (i.e., fish) and terrestrial (i.e., birds) predators (Davis & Huber, 2007) by hiding and defending their shelters. In the pelagic habitat, streamlined individuals with more elongated abdomen might be more efficient to move in the muddy substrate and escape from predators via tail flipping (Patullo & MacMillan, 2004; Wine & Krasne, 1972). Furthermore, streamlined bodies with thicker chelae might provide a defense against predators through gape limitation (Davis & Huber, 2007; Englund & Krupa, 2000; Garvey et al., 2003). As the extent of morphological differentiation between habitats differed between lakes (Appendix S4), it would be interesting to determine whether the extent of differences in environmental conditions between the littoral and the pelagic habitats drives the intensity of the observed morphological differentiations.

As expected, littoral individuals consumed more resources originating from the littoral habitat than pelagic individuals. Given the turnover rate of stable isotope values in crayfish muscle tissue (>1 month; Carolan, Mazumder, Dimovski, Diocares, & Twining, 2012; Glon, Larson, & Pangle, 2016), these findings indicate that the feeding activity of individuals occurs within their respective habitats. There was, however, no significant difference in trophic position between littoral and pelagic individuals. Trophic positions of 3 indicated that *P. clarkii* feed on more than one trophic level and are thus omnivorous, as observed previously in the studied ecosystems (Jackson et al., 2017). This omnivorous diet was likely composed of a mixture of primary producers, invertebrates, and fish (eggs and larvae or carrion) in both habitats (Gutiérrez‐Yurrita et al., 1998). Although it remains to be quantified, these results suggest that, in each habitat, *P. clarkii* display an opportunistic foraging strategy with a diet being primarily driven by resource availability rather than a form of trophic specialization that varies between habitats.

Phenotypic differentiation between littoral and pelagic individuals was not associated with genetic differentiation, highlighting the existence of nonassortative mating. The absence of significant genetic differentiation might be due to the recent colonization of lakes by *P. clarkii* (<60 years, recent population bottleneck event; Dlugosch & Parker, 2008) and/or low reproductive isolation due to the relatively small size of the studied lakes (mean ± *SE* = 13.90 ± 1.78 ha). Thus, our results demonstrate that, within each lake, *P. clarkii* display a stable resource polymorphism with high gene flow between morphs and form a unique population (Smith & Skúlason, 1996). However, the temporal dynamic of this resource polymorphism remains to be quantified because gene flow between morphs could be reduced (e.g., philopatry behavior, breeding temporal segregation, emerging differences in mate choice), thus increasing the genetic differentiation along the littoral–pelagic gradient (Meyer, 1990). In general, littoral–pelagic divergences observed in fish species are explained by combination of both phenotypic plasticity and genetic differences (Faulks et al., 2015; Komiya et al., 2011; Smith & Skúlason, 1996). Here, trophic differentiation was not different from what was expected under the drift hypothesis (*P*_ST_ < *F*_ST_), but *P*_ST_ value for morphology was significantly higher than the *F*_ST_. Thus, adaptive process could explain the divergence of morphology, but this needs further investigations. Therefore, future studies should explore the relative importance of selection versus phenotypic plasticity in driving phenotypic variation within invasive species.

In conclusion, we showed that stable resource polymorphism occurred between littoral and pelagic individuals of a recent biological invasion (Smith & Skúlason, 1996). The establishment of resource polymorphism within invasive populations can have important ecological and evolutionary implications, such as leading to different ecological impacts on the littoral and pelagic food chains (Ruokonen et al., 2012; Vander Zanden & Vadeboncoeur, 2002). Ecosystem impacts of invasive crayfish can vary among their populations (Evangelista, Lecerf, et al., 2019), and the present study suggests that they could also depend on within‐population characteristics.

## CONFLICT OF INTEREST

The authors declare no conflict of interest.

## AUTHOR CONTRIBUTION

JC, CE, IL, and GL designed the study; CE and JC collected the data; RE, IL, and GL performed the genetic analyses; IL and CE analyzed the data; IL drafted the first version of the manuscript; and CE, RE, GL, and JC contributed to revisions. All authors approved the final version of the manuscript.

## Data Availability

All data used in this study are available on Dryad at https://doi.org/10.5061/dryad.m905qftxg.
