# Supplementary material for: Stable resource polymorphism along the benthic littoral–pelagic axis in an invasive crayfish
Source: Ecol Evol. 2020 Feb 14;10(5):2650–60. doi: 10.1002/ece3.6095 (PMC7069303; doi:10.1002/ece3.6095)
Supplement: Supplementary file 1 [file ECE3-10-2650-s001.docx]

**Appendix**

**Appendix S1** Environmental characteristics of the 16 sampled lakes (Haute-Garonne, France). Resource polymorphism was studied in 7 lakes (highlighted in bold) where sufficient number of *Procambarus clarkii* were collected in both habitats (mean number of individuals per habitat = 19.79 ± 0.8 SD). Distance pelagic traps was assessed by measuring the distance (in meters) perpendicularly to the shore with GIS (accuracy of GPS ± 5m). Predation pressure was not assessed in lake O this year.

| **Lake** | **Latitude (N)** | **Longitude (E)** | **Invasion date** | **Surface (m^2^)** | **Proportion of littoral habitat (%)**  **(depth <3 m)** | **Max depth (m)** | **Depth pelagic traps (m)** | **Pelagic traps distances to the shore ( GPS accuracy ± 5 m)** | **Predation (BPUE fish g.gillnet^-1^.hr^-1^)** | **Littoral abundance (CPUE *P. clarkii* ind.trap^-1^.hr^-1^)** | **Pelagic abundance (CPUE *P. clarkii* ind.trap^-1^.hr^-1^)** |
| --- | --- | --- | --- | --- | --- | --- | --- | --- | --- | --- | --- |
| **A** | 43.322349 | 1.202065 | 1995 | 91044 | 42 | 5.00 | 4.76 | 71.72 | 654.91 | 3.21 | 1.17 |
| **B** | 43.3171547 | 1.1989507 | 2000 | 92217 | 20 | 6.10 | 4.96 | 43.67 | 1109.721 | 2.96 | 3.92 |
| C | 43.530329 | 1.289764 | 1995 | 203628 | 100 | 2.90 | 1.82 | 89.83 | 318.26 | 1.38 | 0.17 |
| D | 43.453752 | 1.27367 | 1995 | 271400 | 48 | 5.60 | 1.93 | 42.10 | 18.43 | 0.00* | 0.00 |
| E | 43.519202 | 1.354537 | 1995 | 18895 | 100 | 1.90 | 1.57 | 82.33 | 453.12 | 0.00* | 0.00 |
| F | 43.506265 | 1.337336 | 1995 | 213016 | 84 | 3.50 | 2.91 | 77.50 | 2071.40 | 1.79 | 0.00 |
| **G** | 43.386392 | 1.265891 | 1995 | 182103 | 65 | 3.70 | 2.80 | 65.77 | 829.92 | 2.83 | 1.71 |
| H | 43.343186 | 1.22705 | 1995 | 43951 | 80 | 3.70 | 3.25 | 75.00 | 2719.99 | 2.25 | 1.00 |
| **I** | 43.3208803 | 1.1948792 | 2007 | 131679 | 34 | 5.00 | 4.23 | 80.40 | 1035.88 | 2.54 | 2.21 |
| **J** | 43.3719279 | 1.258473 | 1996 | 103909 | 44 | 4.80 | 3.58 | 53.31 | 0 | 2.17 | 1.08 |
| **K** | 43.364617 | 1.251335 | 1997 | 162864 | 55 | 5.10 | 3.56 | 72.60 | 617.45 | 7.13 | 10.63 |
| L | 43.205616 | 1.040017 | 1995 | 87037 | 34 | 5,90 | 5.16 | 85.77 | 1166.15 | 2.92 | 0.38 |
| **M** | 43.207631 | 1.046572 | 1995 | 210002 | 39 | 6.90 | 5.33 | 139.40 | 745.54 | 4.79 | 2.63 |
| N | 43.208923 | 1.038937 | 1998 | 145985 | 34 | 7.00 | 5.15 | 86.50 | 1958.70 | 0.00* | 0.63 |
| O | 43.551857 | 1.261958 | 1995 | 6961 | 64 | 3.90 | 3.16 | 22.33 | NA | 0.00* | 0.00 |
| P | 43.551353 | 1.259427 | 1995 | 28332 | 61 | 4.30 | 3.25 | 48.00 | 109.00 | 0.38 | 0.13 |

* *P. clarkii* was present in the lake and collected using complementary sampling methods but not were sampled in the traps.

**Appendix S2** List of the 14 microsatellites amplified in three PCR multiplex. All primers at 10µM each. Selection based on Belfiore & May (2000) and Jiang et al., (2015).

**Multiplex 1**

| **Locus** | **GenBank accession number** | **Allele size range (bp)** | **Forward Primer (5’-3’)** | **Reverse Primer (3’-5’)** | **Fluorescent dye** |
| --- | --- | --- | --- | --- | --- |
| PclG-15 | AF290927 | 120-200 | GGC GTG ACG CCA ACG TGT CTT | GGC TGG CCA CTT TGT TAG CCT GAG | ATTO 550 |
| PclG-27 | AF290932 | 100-130 | AAT CTT AAG ATC ATG AAA AAG GTA | TTT AAG GAA CGT ATA AGA AAA GAC | FAM |
| PclG-16 | AF290928 | 80-180 | CTC GGA ATG TCC ACC TGA GA | TCA TTA TGG ATT TTG TCA ATC TAT | HEX |
| PclG-04 | AF290921 | 170-255 | TAT ATC AGT CAA TCT GTC CAG | TCA GTA AGT AGA TTG ATA GAA GG | FAM |

| **PCR MIX** | **Volume (µL) x1** |
| --- | --- |
| H_2_O | 2.1 |
| Qiagen multiplex PCR Master Mix | 5 |
| DNA | 2 |
| PclG-15 F | 0.1 |
| PclG-15 R | 0.1 |
| PclG-27 F | 0.15 |
| PclG-27 R | 0.15 |
| PclG-16 F | 0.1 |
| PclG-16 R | 0.1 |
| PclG-04 F | 0.1 |
| PclG-04 R | 0.1 |

**Multiplex 2**

| **Locus** | **GenBank accession number** | **Allele size range (bp)** | **Forward Primer (5’-3’)** | **Reverse Primer (3’-5’)** | **Fluorescent dye** |
| --- | --- | --- | --- | --- | --- |
| PclG-29 | AF290934 | 159-210 | GAA AGT CAT GGG TGT AGG TGT AAC | TTT TTG GGC TAT GTG ACG AG | ATTO 550 |
| PclG-07 | AF290922 | 112-124 | CCT CCC ACC AGG GTT ATC TAT TCA | GTG GGT GTG GCG CTC TTG TT | FAM |
| PclG-28 | AF290933 | 238-266 | CTC GGC GAG TTT ACT GAA AT | AGA AGA AAG GGA TAT AAG GTA AAG | HEX |
| PclG-32 | AF290935 | 173-221 | CCC CCA CTC GTC TCT GTG TAT G | TGT GCT TGC GGG AGT GAG C | FAM |
| PCSH0038 | KJ607979 | 150-190 | CAG AGC ACT GTT TGC TAG TGT GT | GCT TCC TCT GTT ATT CAT CAT GC | HEX |

| **PCR MIX** | **Volume (µL) x1** |
| --- | --- |
| H_2_O | 2 |
| Qiagen multiplex PCR Master Mix | 5 |
| DNA | 2 |
| PclG-29 F | 0.1 |
| PclG-29 R | 0.1 |
| PclG-07 F | 0.1 |
| PclG-07 R | 0.1 |
| PclG-28F | 0.1 |
| PclG-28 R | 0.1 |
| PclG-32 F | 0.1 |
| PclG-32 R | 0.1 |
| PCSH0038 F | 0.1 |
| PCSH0038 R | 0.1 |

**Multiplex 3**

| **Locus** | **GenBank accession number** | **Allele size range (bp)** | **Forward Primer (5’-3’)** | **Reverse Primer (3’-5’)** | **Fluorescent dye** |
| --- | --- | --- | --- | --- | --- |
| PclG-48 | AF290941 | 145-175 | CTG TTG GTG ATT TCC GTC AAT TTT | AGA TTC AAC GCT GTG TTC CTG ATC | ATTO 550 |
| PclG-17 | AF290929 | 159-184 | GTC GGG AAC CTA TTT ACA GTG TAT | AAG AGC GAA GAA AGA GAT AAA GAT | HEX |
| PCSH0089 | KJ607988 | 80-120 | GTA TAC ACA GCT TTG GAA CTG GG | GCT TCC TCT GTT ATT CAT CAT GC | HEX |
| PCSH0006 | KP675956 | 140-180 | GGC CAA AAT GTG AAG AGT TGT TA | GAA CCA GAT CAG TGT CAT GTG AG | FAM |
| PCSH0005 | KP675955 | 110-135 | AAC AGA GTG GCA AGG TAC TTG AA | GGC TGT CAC TCG TGT CTT TAG TT | FAM |

| **PCR cycling conditions** | | |
| --- | --- | --- |
| 95°C  94°C  56°C  72°C  60°C  10°C | 15 min  30 s  90 s  60 s  45 min  ∞ | 35 cycles |

| **PCR MIX** | **Volume (µL) x1** |
| --- | --- |
| H_2_O | 2 |
| Qiagen multiplex PCR Master Mix | 5 |
| DNA | 2 |
| PclG-48 F | 0.1 |
| PclG-48 R | 0.1 |
| PclG-17 F | 0.1 |
| PclG-17 R | 0.1 |
| PCSH0089 F | 0.1 |
| PCSH0089 R | 0.1 |
| PCSH0006 F | 0.1 |
| PCSH0006 R | 0.1 |
| PCSH0005 F | 0.1 |
| PCSH0005 R | 0.1 |


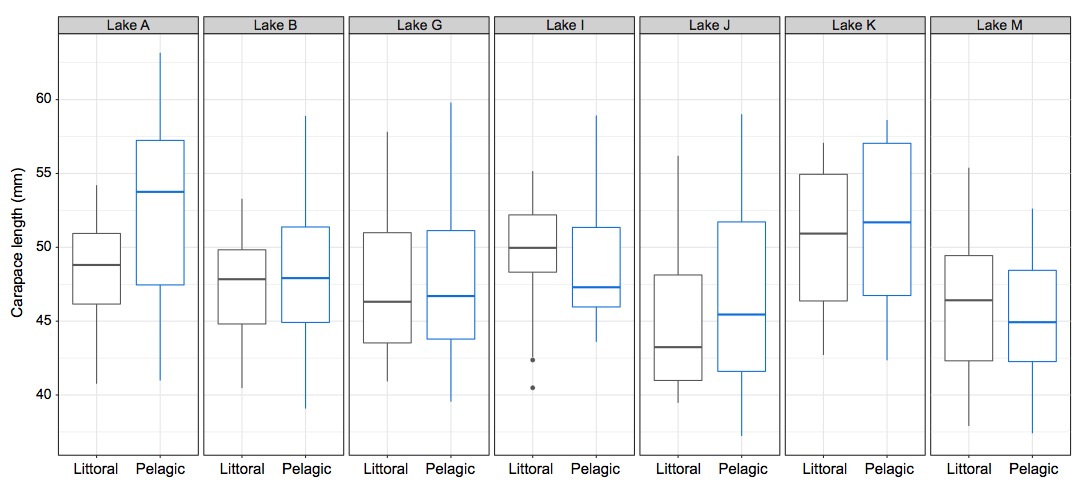


**Appendix S3** Carapace length (mm) of crayfish from littoral (dark grey) and pelagic (blue) habitats in the 7 gravel pit lakes. The boxplot indicates the median (horizontal bar), the first and third quartiles (box), and the maximum and the minimum values no further than 1.5 x inter-quartile range (whiskers). Dots represent outliers.





**Appendix S4** Frequency distribution of *Procambarus clarkii* body morphological scores obtained using a Discriminant Functional Analysis along the littoral-pelagic axis (n_littoral_ = 20, dark grey; n_pelagic_ = 20 except for lake J where n_pelagic_ = 17, blue) of the 7 gravel pit lakes. Note that the X-axis scale is different for Lake K.

**Appendix S5** Stable isotopes values (δ^15^N and δ^13^C; ‰) of individuals *Procambarus clarkii* (circles) from littoral (dark grey) and pelagic (blue) habitats in the 7 gravel pit lakes. Black triangles and white squares represent periphyton and zooplankton in each lake, respectively. Note that the Y-axis scale for Lake K differs from the others.
